# Perinatal mortality, severe maternal morbidity and maternal near miss: protocol of a study integrated with the *Birth in Brazil II* survey

**DOI:** 10.1590/0102-311XEN248222

**Published:** 2024-04-29

**Authors:** Rosa Maria Soares Madeira Domingues, Marcos Augusto Bastos Dias, Marcos Nakamura-Pereira, Rodolfo de Carvalho Pacagnella, Sônia Lansky, Silvana Granado Nogueira da Gama, Ana Paula Esteves-Pereira, Sônia Azevedo Bittencourt, Mariza Miranda Theme, Barbara Vasques da Silva Ayres, Márcia Leonardi Baldisserotto, Tatiana Henriques Leite, Maria do Carmo Leal

**Affiliations:** 1 Instituto Nacional de Infectologia Evandro Chagas, Fundação Oswaldo Cruz, Rio de Janeiro, Brasil.; 2 Instituto Nacional de Saúde da Mulher, da Criança e do Adolescente Fernandes Figueira, Fundação Oswaldo Cruz, Rio de Janeiro, Brasil.; 3 Faculdade de Ciências Médicas, Universidade Estadual de Campinas, Campinas, Brasil.; 4 Secretaria Municipal de Saúde de Belo Horizonte, Belo Horizonte, Brasil.; 5 Escola Nacional de Saúde Pública Sergio Arouca, Fundação Oswaldo Cruz, Rio de Janeiro, Brasil.; 6 Universidade do Estado do Rio de Janeiro, Rio de Janeiro, Brasil.

**Keywords:** Perinatal Mortality, Morbidity Surveys, Pregnancy, Puerperium, Mortalidade Perinatal, Inquéritos de Morbidade, Gravidez, Puerpério, Mortalidad Perinatal, Encuestas de Morbilidad, Embarazo, Puerperio

## Abstract

Brazil presents high maternal and perinatal morbidity and mortality. Cases of severe maternal morbidity, maternal near miss, and perinatal deaths are important health indicators and share the same determinants, being closely related to living conditions and quality of perinatal care. This article aims to present the study protocol to estimate the perinatal mortality rate and the incidence of severe maternal morbidity and maternal near miss in the country, identifying its determinants. Cross-sectional study integrated into the research *Birth in Brazil II*, conducted from 2021 to 2023. This study will include 155 public, mixed and private maternities, accounting for more than 2,750 births per year, participating in the *Birth in Brazil II* survey. We will collect retrospective data from maternal and neonatal records of all hospitalizations within a 30-day period in these maternities, applying a screening form to identify cases of maternal morbidity and perinatal deaths. Medical record data of all identified cases will be collected after hospital discharge, using a standardized instrument. Cases of severe maternal morbidity and maternal near miss will be classified based on the definition adopted by the World Health Organization. The perinatal deaths rate and the incidence of severe maternal morbidity and maternal near miss will be estimated. Cases will be compared to controls obtained in the *Birth in Brazil II* survey, matched by hospital and duration of pregnancy, in order to identify factors associated with negative outcomes. Results are expected to contribute to the knowledge on maternal morbidity and perinatal deaths in Brazil, as well as the development of strategies to improve care.

## Resumo

O Brasil apresenta elevada morbimortalidade materna e perinatal. Casos de morbidade materna grave, *near miss* materno e óbitos perinatais são indicadores importantes de saúde e compartilham dos mesmos determinantes sociais, tendo estreita relação com as condições de vida e qualidade da assistência perinatal. Este artigo pretende apresentar o protocolo de estudo que visa estimar a taxa de mortalidade perinatal e a incidência de morbidade materna grave e *near miss* materno no país, assim como identificar seus determinantes. Trata-se de estudo transversal integrado à pesquisa *Nascer no Brasil II*, realizada entre 2021 e 2023. Serão incluídas neste estudo 155 maternidades públicas, mistas e privadas, com mais de 2.750 partos por ano, participantes do *Nascer no Brasil II*. Nessas maternidades, será realizada coleta retrospectiva de dados de prontuário materno e neonatal de todas as internações ocorridas num período de 30 dias, com aplicação de uma ficha de triagem para identificação de casos de morbidade materna e de óbito perinatal. Dados de prontuário de todos os casos identificados serão coletados após a alta hospitalar, utilizando instrumento padronizado. Casos de morbidade materna grave e *near miss* materno serão classificados por meio da definição adotada pela Organização Mundial da Saúde. Será estimada a taxa de mortalidade perinatal e a incidência de morbidade materna grave e *near miss* materno. Os casos serão comparados a controles obtidos na pesquisa *Nascer no Brasil II*, pareados por hospital e duração da gestação, visando a identificação de fatores associados aos desfechos negativos. Espera-se que os resultados deste artigo contribuam para o conhecimento sobre a morbidade materna e a mortalidade perinatal no país, bem como para a elaboração de estratégias de melhoria do cuidado.

## Resumen

Brasil tiene una alta morbimortalidad materna y perinatal. Los casos de morbilidad materna severa, maternal *near miss* y muertes perinatales son importantes indicadores de salud y comparten los mismos determinantes sociales, y tienen una estrecha relación con las condiciones de vida y la calidad de la asistencia perinatal. Este artículo pretende presentar el protocolo de estudio que tiene como objetivo estimar la tasa de mortalidad perinatal y la incidencia de morbilidad materna severa y maternal *near miss* en el país, así como identificar sus determinantes. Se trata de un estudio transversal integrado a la investigación *Nacer en Brasil II*, realizada entre el 2021 y el 2023. Este estudio incluirá 155 maternidades públicas, mixtas y privadas, con más de 2.750 partos al año, que participan en el *Nacer en Brasil II*. En estas maternidades, se realizará una recopilación retrospectiva de datos de las historias clínicas maternas y neonatales de todas las hospitalizaciones ocurridas en un período de 30 días, con la aplicación de un formulario de triaje para identificar casos de morbilidad materna y de muerte perinatal. Los datos de las historias clínicas de todos los casos identificados se recopilarán tras el alta hospitalaria, mediante un instrumento estandarizado. Los casos de morbilidad materna severa y maternal *near miss* se clasificarán por medio de la definición adoptada por la Organización Mundial de la Salud. Se estimará la tasa de mortalidad perinatal y la incidencia de morbilidad materna severa y maternal *near miss*. Los casos se compararán con los controles obtenidos en el estudio *Nacer en Brasil II*, emparejados por hospital y duración del embarazo, para identificar factores asociados con desenlaces negativos. Se espera que los resultados de este artículo contribuyan al conocimiento sobre la morbilidad materna y la mortalidad perinatal en el país, así como a la elaboración de estrategias para mejorar el cuidado.

## Introduction

Maternal, early neonatal and fetal mortality are important health indicators and share the same determinants. In Brazil, such deaths are still unacceptably high and associated with socioeconomic inequalities and failures in the care for pregnant women, childbirth, and the newborn ^1^.

The maternal mortality ratio (MMR) showed a decline in the period 1990-2001 in Brazil, remaining stable at around 60 deaths/100,000 live births until 2019 ^2^. In 2020, it increased due to the COVID-19 pandemic, reaching more than 100/100,000 live births in 2021 ^3^. However, even in the pre-pandemic period, the value recorded in the country was much higher than the Sustainable Development Goal target of less than 30 per 100,000 live births in 2030 ^4^ set for Brazil.

Although the MMR is high, maternal death is a rare event. That is why the study of severe maternal morbidity has been recommended, defined as the occurrence of a severe maternal complication during pregnancy, delivery, or puerperium (up to 42 days after the end of pregnancy). The term maternal near miss is used for women who almost died but survived complications that occurred during pregnancy and childbirth ^5^ ^,^ ^6^.

Cases of maternal near miss are at the extreme of severe maternal morbidity gravity and share the same problems and obstacles associated with care during pregnancy, delivery and puerperium with maternal deaths, thus can be used as a sentinel event ^7^. As they are more frequent than maternal deaths, maternal near miss cases allow more robust evaluation of the determinants of maternal death and the quality of obstetric care ^7^ ^,^ ^8^.

In 2009, the World Health Organization (WHO) proposed a maternal near miss classification based on organ dysfunction criteria, seeking an international parameter standardization that would allow comparability among different studies, institutions, and countries ^6^. In Brazil, local ^9^ ^,^ ^10^ and national ^11^ ^,^ ^12^ studies estimated the maternal near miss ratio using the WHO criteria from medical record collection data. Pacagnella et al. ^11^, in a multicenter study conducted in 2010/2011 in 28 maternities, estimated an incidence of maternal near miss of 9.5/1,000 live births, while Dias et al. ^12^, using data from the *Birth in Brazil I* survey conducted in 2011/2012, estimated an incidence of 10.2 cases of maternal near miss per 1,000 live births. However, monitoring maternal morbidity through existing information systems is limited due to doubts on the validity of available information in the Brazilian Unified National Health System (SUS) Hospital Information System (SIH/SUS) ^13^, the only system that has morbidity data, therefore, severe maternal morbidity and maternal near miss estimates depend on specific studies.

Perinatal mortality includes fetal deaths with ≥ 22 gestational weeks and/or weight ≥ 500g and deaths of live births with any weight or gestational age occurring up to the sixth day of life. Therefore, this indicator reflects the quality of obstetric and perinatal care, and its analysis is relevant for the identification of preventive actions that allow the achievement of mutual gains in the reduction of preventable early fetal and neonatal deaths.

Globally, it is estimated that 45% of deaths under five years of age occur in the neonatal period, corresponding to the loss of 2.6 million lives per year. In addition, about 2.1 million fetal deaths occur in the last three months of gestation or at the time of delivery. The reduction in neonatal mortality and fetal mortality worldwide was less than the reduction in under-five mortality in the period 1990-2015 ^14^. In Brazil, perinatal mortality also showed smaller reduction than infant mortality in the 1982-2015 period, being estimated at 15.5 per 1,000 live births in 2018 ^15^.

Brazilian estimates mask differences in the quality of data regarding vital events, varying substantially among states and are due to underreporting of births and deaths, or inaccuracies in the classification of the cause of death. For perinatal mortality, there are additional challenges due to errors in the classification of live births, stillbirths, and abortions, with invasion and/or evasion of deaths and births, in addition to high data incompleteness and errors in the classification of the underlying cause of death, often incorrectly identified ^16^ ^,^ ^17^ ^,^ ^18^ ^,^ ^19^. Failures in vital records result in gaps in the understanding of the determinants of perinatal mortality, impairing the definition of priorities, guidelines, and policies, and thus the effectiveness of actions to prevent and control the occurrence of deaths.

The WHO recommends that the review of maternal and perinatal deaths be performed globally in all hospitals, this audit being relevant to the objectives of the *Every Newborn Action Plan* (ENAP), a global plan aimed at eliminating preventable fetal and neonatal mortality and reduction of maternal morbidity and mortality ^20^. However, there are barriers to achieve it, the main ones being the time required to perform audits, lack of staff training, and incomplete or insufficient data ^21^. In addition, operational research is needed to evaluate the most cost-effective strategies for implementing the review of maternal and perinatal deaths in low- and middle-income countries ^22^ ^,^ ^23^.

Considering the need of updated data to allow the evaluation of severe maternal morbidity and perinatal mortality in Brazil and the development of strategies to improve obstetric and neonatal care, this article aims at presenting the protocol of the study to evaluate severe maternal morbidity and perinatal mortlity in Brazil. Our hypothesis is that severe maternal morbidity and perinatal mortality are associated with women’s socioeconomic conditions, such as race/skin color, education, and income, as well as with timely access to prenatal care, delivery, and neonatal care services, and that there is an association between severe maternal morbidity and perinatal mortality.

## Methods

This is a cross-sectional hospital-based study with national coverage, integrated with *Birth in Brazil II: National Research on Abortion, Labor and Childbirth* (*Birth in Brazil II*) in the period 2021-2023.

### *Birth in Brazil II* survey

The *Birth in Brazil II* survey protocol is published in Leal et al. ^24^. Briefly, it is a national hospital-based survey with a planned sample of 22,050 women hospitalized for childbirth and approximately 2,205 women hospitalized for abortion care in 465 health facilities with 100 or more deliveries per year. The sample was stratified by macroregion of Brazil (North, Northeast, Southeast, South, Central-West), type of hospital (public/mixed/private) and location (capital city and cities located in the metropolitan area/other cities). In hospitals with 500 or more births per year, 50 postpartum women will be interviewed, while in hospitals with 100-499 births per year, 30 postpartum women will be interviewed. The number of interviews with miscarriage puerperae will vary among hospitals, corresponding to the number of hospitalizations for miscarriage that occurred until the expected sample of postpartum women in each hospital is reached.

In each hospital, the following will be considered eligible: puerperae from hospital deliveries with live births of any weight or gestational age; puerperae from hospital deliveries of fetal deaths with gestational age ≥ 22 weeks or weight ≥ 500g; and women admitted with a diagnosis of miscarriage.

Eligibility criteria will exclude: women who delivered in another health institution, at home, or on public roads; women hospitalized with a diagnosis of miscarriage but discharged while still pregnant; women who did not speak Portuguese; women with hearing impairment or severe mental disability; and women hospitalized for delivery by court order.

For all women included in the study, interviews will be conducted in the immediate puerperium; prenatal cards will be photographed, when available, for later data extraction; and clinical data from hospital records will be extracted after discharge or on the 42nd day of puerperium, in the case of prolonged hospitalizations of puerperae, or on the 28th day of life, in the case of prolonged hospitalization of the newborn.

Two telephone interviews, at two and four months after delivery/abortion, will be conducted to assess utilization of services after discharge, late maternal and neonatal morbidity, breastfeeding, maternal mental health, and mistreatment in labor and abortion care.

In all health units, a data collection instrument will also be applied to the manager, developed based on current legislation, to get to know the hospital’s structure.

The *Birth in Brazil II* survey was approved by the Brazilian National Research Ethics Committee (CONEP; CAAE: 21633519.5.0000.5240), on March 11, 2020 (n. 3,909,299), with approval from the institutional review board or from the clinical board when the local committees are absent.

### The *Severe Maternal Morbidity* and *Perinatal Mortality* studies: integrated studies of the *Birth in Brazil II* survey

#### Objectives of the *Severe Maternal Morbidity* and *Perinatal Mortality* studies

The *Severe Maternal Morbidity* and *Perinatal Mortality* studies aim to: (i) estimate the rate of perinatal mortality and the ratio of severe maternal morbidity and maternal near miss with the description of their causes; (ii) investigate the socioeconomic, demographic and obstetric characteristics associated with severe maternal morbidity, maternal near miss and perinatal death; (iii) investigate the association between prenatal care conditions, maternity hospital structure, and processes and procedures in labor, birth, and puerperium care with severe maternal morbidity, maternal near miss, and perinatal death; (iv) investigate the association between severe maternal morbidity/maternal near miss and perinatal mortality; and (v) validate the causes of perinatal death after investigation of fetal and neonatal deaths by trained obstetricians and pediatricians.

#### Sample design

All hospitals included in the *Birth in Brazil II* survey that had more than 2,750 deliveries per year were considered eligible for the *Severe Maternal Morbidity* and *Perinatal Mortality* studies. This definition was based on the number of live births in the hospitals included in the *Birth in Brazil II* sample, these hospitals being in the upper tercile regarding the number of births. Approximately 46.4% of live births in Brazil occur in hospitals with more than 2,750 live births per year. The choice of hospitals with a higher number of births was due to the low frequency of the severe maternal morbidity and perinatal death outcomes, with higher occurrence expected in hospitals with a large volume of deliveries, which in general are reference for high-risk pregnancies ^25^. In the selected hospitals, considering an estimate of 10% of severe maternal morbidity cases, 1% of maternal near miss and 1% of perinatal mortality, it is expected to identify 5,800 cases of severe maternal morbidity, 580 cases of maternal near miss and 580 cases of perinatal death from the review of 58,000 hospital admissions. Table 1 presents the hospitals included according to type of unit, macroregion, and location.

**Table 1:** Absolute and relative sample size of hospitals by sampling stratum.

Sample stratum	Macroregion	Location	Hospitals (≥ 2,750 deliveries/year)			Sample *Birth in Brazil II* **	**% (sample *Severe Maternal Morbidity and Perinatal Mortality*/ sample *Birth in Brazil II*)**
			Universe *	**Sample *Severe Maternal Morbidity and Perinatal Mortality***	% (sample/ universe)		
Public hospitals							
1111	North	Metropolitan Region	14	11	78.6	13	84.6
1211	North	Non-Metropolitan Region	3	2	66.7	11	18.2
2111	Northeast	Metropolitan Region	35	18	51.4	25	72.0
2211	Northeast	Non-Metropolitan Region	15	5	33.3	18	27.8
3111	Southeast	Metropolitan Region	53	21	39.6	34	61.8
3211	Southeast	Non-Metropolitan Region	5	3	60.0	9	33.3
4111	South	Metropolitan Region	9	6	66.7	10	60.0
4211	South	Non-Metropolitan Region	2	2	100.0	3	66.7
5111	Central-West	Metropolitan Region	11	6	54.5	9	66.7
5211	Central-West	Non-Metropolitan Region	1	1	100.0	4	25.0
Mixed hospitals							
1121	North	Metropolitan Region	6	6	100.0	7	85.7
1221	North	Non-Metropolitan Region	2	2	100.0	5	40.0
2121	Northeast	Metropolitan Region	19	9	47.4	16	56.3
2221	Northeast	Non-Metropolitan Region	9	5	55.6	16	31.3
3121	Southeast	Metropolitan Region	17	10	58.8	21	47.6
3221	Southeast	Not Metropolitan Region	10	6	60.0	26	23.1
4121	South	Metropolitan Region	15	6	40.0	19	31.6
4221	South	Non-Metropolitan Region	2	2	100.0	11	18.2
5121	Central-West	Metropolitan Region	3	3	100.0	6	50.0
5221	Central-West	Non-Metropolitan Region	2	2	100.0	7	28.6
Private hospitals							
1131	North	Metropolitan Region	3	2	66.7	8	25.0
1231	North	Non-Metropolitan Region	0	0	-	2	0.0
2131	Northeast	Metropolitan Region	8	4	50.0	23	17.4
2231	Northeast	Non-Metropolitan Region	0	0	-	3	0.0
3131	Southeast	Metropolitan Region	23	15	65.2	53	28.3
3231	Southeast	Non-Metropolitan Region	1	1	100.0	10	10.0
4131	South	Metropolitan Region	6	5	83.3	17	29.4
4231	South	Non-Metropolitan Region	0	0	-	3	0.0
5131	Central-West	Metropolitan Region	4	2	50.0	13	15.4
5231	Central-West	Non-Metropolitan Region	0	0	-	3	0.0
Total	-		278	155	55.8	405	38.3

Metropolitan Region: capital and municipality located in Metropolitan Region; non-Metropolitan Region: other municipalities.

* Universe of hospitals with ≥ 2,750 deliveries/year according to data from Brazilian Information System on Live Births (SINASC-2017);

** Sample of the 405 hospitals in the *Birth in Brazil II* survey with 500 or more deliveries/year. The remaining 60 hospitals sampled in *Birth in Brazil II* with 100-499 deliveries/year belong to other sampling strata (two in each sampling stratum, except North/Metropolitan Region/Mixed stratum, which has no hospital and the Southeast/Metropolian Region/Mixed and South/ Metropolitan Region/Mixed strata with three hospitals each).

#### Eligibility criteria

All women and newborns aged under seven days of life admitted to the hospital are considered eligible for the *Severe Maternal Morbidity* and *Perinatal Mortality* studies, regardless of the reason for admission; the place of delivery/abortion (at the institution, at another institution, on public roads, at home); the pregnancy outcome (abortion, live birth, stillbirth, discharged still pregnant); and the type of discharge. The differences in relation to the *Birth in Brazil II* eligibility criteria are due to the non-conduct of interviews with postpartum women, which resulted in the non-exclusion of women who did not speak Portuguese, who had a hearing impairment or severe mental disability, or who were admitted for childbirth by judicial order. On the other hand, women who gave birth at home or on public roads, or who were transferred from another health institution were considered eligible for the *Severe Maternal Morbidity* and *Perinatal Mortality* studies, aiming to capture situations of greater morbidity that could be presented by these women.

#### Data collection

In each hospital, a retrospective collection of medical record data (physical or electronic, according to availability in each facility) is being carried out for all obstetric and neonatal admissions during a period of 30 calendar days, with a start and end date defined by the research team for each hospital.

Before the beginning of the field work in each hospital, an interview is held with the manager of the health facility or a professional indicated by the manager, for the recognition of the recording instruments used by the service in the identification of hospital admissions of women and of newborns, as well as of fetal and neonatal deaths. The fieldwork period starts at 0:00a.m. of the first day defined for each unit, ending at midnight of the 30th consecutive day.

For each hospitalization identified in the study period, a triage form is filled out, based on data from the women’s medical records (to identify maternal morbidity and fetal deaths) or from the newborns (to identify neonatal deaths). Cases of maternal death were also included in this screening step.

Maternal data obtained in this screening instrument include the criteria proposed by the WHO for maternal morbidity surveillance ^6^ ^,^ ^26^, as well as the criteria previously used by the National Severe Maternal Morbidity Surveillance Network ^27^, which include maternal conditions and additional procedures. The use of expanded criteria in the screening stage was intended to minimize the risk of losing maternal morbidity cases. Box 1 describes the criteria used in the screening process.

**Box 1:** List of conditions used for maternal morbidity surveillance.

HYPERTENSIVE COMPLICATIONS	HEMORRHAGIC COMPLICATIONS	OTHER COMPLICATIONS	INDICATORS OF SEVERITY MANAGEMENT
Severe preeclampsia Eclampsia Severe hypertension HELLP syndrome Hypertensive encephalopathy Fatty liver	Premature placental abruption Placenta accreta/Increta/Percreta Ectopic pregnancy Postpartum/Post abortion hemorrhage Uterine rupture	Pulmonary edema Seizures Sepsis Thrombocytopenia < 100,000 Thyrotoxic crisis Shock Acute respiratory failure Acidosis Heart disease Stroke Coagulation disorders Pulmonary thromboembolism Diabetic ketoacidosis Jaundice/Liver dysfunction Meningitis Acute renal failure Endometritis	Blood product transfusion Central venous access Intensive care unit admission Prolonged hospitalization (> 7 days) Non anesthesia-related intubation Return to the operating room Laparotomy Hysterectomy Use of magnesium sulfate

In all hospitalizations with the identification of at least one condition indicating either maternal morbidity or perinatal death, the complete extraction of data from medical records is performed after hospital discharge or on the 42nd day of maternal puerperium or on the 7th day of birth of the newborn. This data collection aims to identify cases of severe maternal morbidity, maternal near miss and perinatal death, as well as to obtain information on demographic and social characteristics, clinical and obstetric history, current pregnancy data, hospital delivery and abortion care, pregnancy and puerperium complications, and neonatal care.

All data collection is performed using electronic forms inserted in the REDCap system (https://www.redcap.fiocruz.br/redcap/), hosted in the Oswaldo Cruz Foundation (Fiocruz) server. The electronic questionnaires allow internal reviews that reduce the number of typing and filing errors, such as blank spaces or not applicable, as well as the filling of invalid numbers (such as dates, age, gestational age, etc.). In addition, online access to the database allows real-time monitoring of the fieldwork. All data collection is being performed by nurses, most with a specialization in obstetrics, trained by the project team, supervised by the core team.

#### Variables

#### a) Outcome variables

(a) Maternal death: maternal death will be classified as all deaths that occurred during hospitalization during pregnancy, delivery or until the 42nd day postpartum/abortion due to causes that define maternal death according to the International Classification of Diseases, 10th revision (ICD-10) ^28^ ^,^ ^29^. 

(b) severe maternal morbidity: every woman who presents one of the 26 criteria proposed by the WHO for classification of a case of potentially life-threatening condition ^6^ described in Box 2 will be classified as a case of severe maternal morbidity.

**Box 2:** Criteria for defining severe maternal morbidity.

HEMORRHAGIC DISORDERS	HYPERTENSIVE DISORDERS	OTHER SYSTEMIC DISORDERS	INDICATORS OF SEVERITY MANAGEMENT
Premature placental detachment Placenta accreta, increta or percreta Ectopic pregnancy Postpartum hemorrhage Uterine rupture	Severe pre-eclampsia Eclampsia Severe hypertension Hypertensive encephalopathy HELLP syndrome	Endometritis Acute pulmonary edema Respiratory failure Seizures Sepsis Shock Thrombocytopenia < 100,000 platelets Thyrotoxic crisis	Blood transfusion Central venous access Hysterectomy Admission to intensive care unit Prolonged hospitalization (> 7 days postpartum) Non-anesthetic intubation Return to surgical center Surgical intervention

(c) Maternal near miss: every woman who presents one of the 25 criteria proposed by the WHO for the classification of maternal near miss based on organ dysfunction ^6^ described in Box 3 and who does not evolve to death during the period of hospitalization will be classified as a case of MNM.

**Box 3:** Criteria for defining a maternal near miss case.

CLINICAL CRITERION	LABORATORY CRITERION	MANAGEMENT CRITERION
Acute cyanosis Gasping Respiratory rate > 40bpm or < 6bpm) Shock Oliguria unresponsive to fluids or diuretics Failure to form clots Prolonged loss of consciousness (≥ 12 hours) Prolonged loss of consciousness and absence of pulse/heartbeat Cerebrovascular accident Uncontrollable seizures/total paralysis Jaundice in the presence of preeclampsia	O_2_ saturation < 90% for ≥ 60 minutes PaO_2_/FiO_2_ < 200 Creatinine ≥ 300µmol/mL or ≥ 3.5mg/dL Bilirubin > 100µmol/L or > 6.0mg/dL pH < 7.1 Lactate > 5 Severe acute thrombocytopenia (< 50,000 platelets) Unconsciousness and presence of glucose and ketones in the urine	Continued use of vasoactive drugs Intubation and ventilation for ≥ 60 minutes not related to anesthesia Hysterectomy due to bleeding or infection Transfusion of ≥ 5 units of whole blood or packed red blood cells Dialysis for acute renal failure Cardiopulmonary resuscitation

PaO_2_/FiO_2_: ratio of oxygen partial pressure in arterial blood to inspiratory fraction of oxygen.

(d) Perinatal death: every fetal death (with weight ≥ 500g or gestational age ≥ 22 weeks) or neonatal death occurring up to the 6th day of life, regardless of gestational age or birth weight.

#### b) Explanatory variables

(a) Demographic and socioeconomic characteristics: maternal age, race/skin color, years of schooling, economic class, paid work, marital status, macroregion of residence;

(b) Clinical and obstetric characteristics: history of chronic disease, obstetric history (total number of pregnancies, deliveries and abortions; previous cesarean section), previous negative outcomes (low birth weight, prematurity, stillbirth, neonatal death);

(c) Current pregnancy characteristics: number of prenatal visits, Robson ^30^ group, mode of delivery, diagnoses of clinical or obstetric pathologies in the current pregnancy;

(d) Characteristics of delivery/abortion care: data on care received (e.g., tests and procedures performed and medications received during abortion, labor and puerperium care; induction of labor; mode of delivery; type of uterine evacuation; surgical interventions; transfusion of blood products), delays in receiving critical interventions, maternal morbidity diagnosed during hospitalization, admission to intensive care unit (ICU);

(e) Characteristics of birth and neonatal care: sex, gestational age at birth, birth weight, first and fifth minute Apgar scores, congenital malformation, pathology diagnoses, data on neonatal care received (e.g., neonatal resuscitation; tests and procedures performed; medications received, such as antibiotics, surfactant, vasoactive drugs; phototherapy; ventilatory support; blood product transfusion; breastfeeding practices), hospitalization in an ICU or neonatal intermediate care unit.

All variables to be used in the analysis will come from the data collection instruments of the study.

### Data analysis

Two strategies will be used, the first descriptive and the second analytical (case-control study).

In the first stage, a descriptive analysis of the cases of severe maternal morbidity, maternal near miss and perinatal death according to cause and maternal characteristics will be performed, as well as an estimate of the indicators described in Box 4, with 95% confidence intervals (95%CI).

**Box 4:** Maternal morbidity and perinatal mortality indicators.

Maternal near miss ratio = number of maternal near miss cases/total live births x 1,000
Maternal mortality ratio = number of maternal deaths per total live births x 100,000
Maternal near miss/maternal mortality ratio = ratio between cases of maternal near miss and maternal death
Severe maternal outcome ratio = number of women with severe maternal outcome (maternal near miss + deaths)/live births x 1,000
Mortality rate = number of maternal deaths/number of maternal deaths + maternal near miss cases x 100
Maternal severity score * = number of organ dysfunction criteria presented by the pregnant/puerperal woman
Maternal severity index * = estimates the probability of death of a woman who presents pregnancy-related complications
Fetal mortality rate: number of fetal deaths/number of fetal deaths + number of live births x 1,000
Early neonatal mortality rate = number of neonatal deaths up to the 6th day of life/total number of live births x 1,000
Perinatal mortality rate = number of fetal deaths + number of early neonatal deaths/number of fetal deaths + number of live births x 1,000

* Souza et al. ^43^.

For perinatal death outcomes, a new Death Certificate (DC) will be independently constructed by two obstetricians (fetal deaths) and two pediatricians (neonatal deaths), containing selected variables such as birth weight, gestational age and causes of death, as well as age, years of schooling and race/color of the mother. Disagreements will be resolved by consensus. After this step, causes of perinatal death will be coded by a professional trained by the Brazilian Center for Disease Classification, identifying and recording the underlying and contributing causes of death, according to the standards of the ICD-10 ^28^ ^,^ ^31^ ^,^ ^32^. A comparative analysis between original and the remade DCs underlying causes and other variables of interest selected in the Brazilian Mortality Information System (SIM) will be conducted by calculating the kappa coefficient and adjusted kappa coefficient for prevalence and evaluating the degree of agreement according to the classification proposed by Landis & Koch ^33^. Considering the remade DC as the new standard, the calculation of sensitivity, specificity, positive or negative predictive value of the underlying causes of perinatal deaths will be conducted ^34^.

For maternal morbidity and perinatal death outcomes, the hospital care received will be evaluated and delays ^11^ ^,^ ^35^ in access to specific procedures indicated during hospitalization will be identified: admission to intensive care unit (need for ventilatory or hemodynamic support); receiving transfusion of blood derivatives (acute anemia with signs of hypovolemia and/or laboratory tests indicative of transfusion); performing emergency cesarean section (according to diagnosis of maternal or fetal intercurrence); performance of uterine evacuation in the case of abortions (according to clinical criteria at hospital admission and pregnancy evolution); performance of surgical procedures (hysterectomy, laparotomy, videolaparoscopy), and use of magnesium sulfate for severe hypertension/eclampsia (according to maternal signs and symptoms described). Whether there has been adequate treatment for the condition as recommended by the Obstetrics Treatise of the Brazilian Federation of Gynecology and Obstetrics Associations (Febrasgo) ^36^ will be evaluated. For the evaluation of delay, the time elapsed between the indication of the procedure and its performance will be considered.

In the second stage of the analysis, case-control studies will be carried out, one for each outcome (severe maternal morbidity, maternal near miss, fetal deaths, neonatal deaths and perinatal deaths), which will allow the identification of associations between the studies outcomes and maternal and neonatal factors.

Cases of severe maternal morbidity, maternal near miss and perinatal deaths will be those identified in the *Severe Maternal Morbidity* and *Perinatal Mortality* studies. Four controls for each case will be selected from the *Birth in Brazil II* survey database, matched according to hospital and duration of gestation. Severe maternal morbidity, maternal near miss and perinatal death cases with a clinical and/or laboratory diagnosis of COVID-19 at the time of hospital admission will be excluded from this analysis, as women diagnosed with COVID-19 were not interviewed in the *Birth in Brazil II* survey because they were in respiratory isolation, therefore excluded from controls. Cases of maternal death will be excluded from cases and controls.

Causal models will be developed for each outcome, based on the scientific literature ^37^ ^,^ ^38^ ^,^ ^39^, in order to choose the minimum set of variables for adjustment. Conditional logistic regression will be performed for each outcome with estimation of the crude and adjusted odds ratios (OR) and respective 95%CI. Specific analysis methods may be used in the analysis of each outcome.

The entire analysis process will be performed using procedures for complex samples, with weighting and calibration of the data and incorporation of the design effect, using SPSS 22.0 software (https://www.ibm.com/).

#### Ethical aspects

As this is a retrospective study, from the collection of medical record data, we requested the signing of the Informed Consent Form (ICF) to be waived, and access to the medical records was authorized by the hospital unit and by the Ethics Research Committee of the Sergio Arouca National School of Public Health/Fiocruz (n. 4,230,028, issued on August 21, 2020; and amendment approved in amendment n. 4,473,968, issued on December 18, 2020). All healthcare facilities signed a consent form to participate in the study. All necessary precautions are being taken to ensure the secrecy and confidentiality of information. Numerical codes are used to identify participants, and analyses conducted in a grouped manner, not allowing the identification of participants or hospital units.

## Discussion

This study will allow us to analyze severe maternal morbidity and perinatal mortality in a national sample of public and private hospitals that account for almost half of all births in Brazil. Compared to the research conducted by the Network for Surveillance of Severe Maternal Morbidity in 2010, this study advances by using a probability sample of hospitals in all regions of the country, whereas the Network’s used a convenience sample. Also, the Network survey was not a case-control study and its analyses generally used women with potentially life-threatening condition as a comparison group to assess the effects and determinants of near miss and maternal death ^40^. As for the *Birth in Brazil I* survey, conducted in 2011/2012 ^12^, this study expands its inclusion criteria by including cases of abortion, deliveries that occurred in public roads, at home, and other health institutions, as well as women hospitalized for complications in pregnancy and the puerperium. In addition, it includes the analysis of severe maternal morbidity and maternal near miss cases.

For both outcomes, a census of hospitalizations during a 30-day period in large hospitals will allow us to minimize registration losses, as well as to identify a significant number of cases, due to the high number of hospitalizations and the concentration of these outcomes in larger and more complex hospitals, which are usually referral units for high-risk pregnancies.

For maternal morbidity, the use of a screening form with an expanded list of morbidity criteria, in addition to those recommended by the WHO, aimed at greater sensitivity in detecting cases. Further comparison with cases confirmed as severe maternal morbidity or maternal near miss will allow refining the instrument for future uses if it resulted in many false-positives. Using the WHO-recommended case definitions of severe maternal morbidity and maternal near miss will also allow comparison of the results with previous national studies as well as international studies that adopt this same classification. The inclusion of women with clinical and/or laboratory diagnosis of COVID-19 in the *Severe Maternal Morbidity* and *Perinatal Mortality* studies will also allow estimation of maternal morbidity associated with SARS-COV-2 infection in the descriptive stage of the analysis.

For perinatal deaths, the evaluation of deaths by trained obstetricians and pediatricians and the reclassification of the type of death (fetal or early neonatal) and its causes will allow validating the information available in the SIM and a better estimate of fetal, early neonatal and perinatal mortality rates, as well as a better assessment of their causes and determinants. The correct classification of fetal and early neonatal deaths will allow, besides the correct estimation of the magnitude of the problem, the improvement of prematurity estimates in the country, which are underreported due to inadequate classification of live and dead births, since fetal death is not considered when calculating this indicator. Another important aspect is the analysis of fetal deaths at less than 28 weeks of gestation, since generally the international production addresses only late fetal deaths occurring after the 28th week of gestation ^41^.

The choice of a case-control study was based on the greater effectiveness of this type of design for the analysis of rare outcomes, and its integrated execution with the *Birth in Brazil II* survey for logistical advantages. The *Severe Maternal Morbidity* and *Perinatal Mortality* studies and the *Birth in Brazil II* survey are being conducted in the same years and use the same instruments for medical record data collection. All hospitals included in the *Severe Maternal Morbidity and Perinatal Mortality* study participate in the *Birth in Brazil II* survey, and selecting controls at the same hospital where cases are identified will allow the selection of controls that reflect the prevalence of the exposures studied, reducing the possibility of bias selection. Likewise, using the same data collection tools, standardized training and supervision of the field teams in both studies will reduce the possibility of differential measurement bias.

As limitations, we highlight the inclusion of only hospitals with more than 2,750 deliveries per year in the study. Although this strategy aimed at greater detection of cases, it is possible that the incidence and profile of the identified cases are different from those in smaller hospitals, limiting the external validity of the results of this type of service. The use of data only from hospital records will not allow an adequate evaluation of the use of health services by women and newborns, including primary care, specialized services and hospital care, limiting the analysis of delays between seeking and obtaining healthcare. The *Birth in Brazil II* survey has some exclusion criteria, such as women with non-hospital deliveries, women who were discharged while still pregnant, or women with cognitive or language difficulties to be interviewed. Although these are infrequent situations, these differences will limit the selection of control for cases that present such characteristics. The pairing of cases and controls cannot be made in time (day/week of hospitalization or birth), since data collection in both this and the *Birth in Brazil II* survey does not occur at the same time interval in each hospital. Thus, it is not possible to exclude the possibility that fluctuations in the supply of hospital beds and/or changes in the care teams may have interfered with the care provided. Maternal deaths were not included in the case-control study because they are infrequent events, and the study design would not allow obtaining an adequate sample. However, a specific study on maternal deaths is also being conducted in an integrated manner with the *Birth in Brazil II*, through a census of maternal deaths that occurred during a two-year period in the hospitals participating in the study ^42^, which will provide important information on maternal mortality in these services. Cases of neonatal near miss, which correspond to severe neonatal morbidity occurring in the first six days of life, were also not evaluated, since complete medical record data were only collected for women with maternal morbidity or for newborns that progressed to neonatal death. Neonatal near miss will be assessed in the *Birth in Brazil II* survey with a planned sample of approximately 22,000 births. Finally, we know that the use of information based on medical records has limitations related to incompleteness of data and non-standardization of clinical records. However, it is the data source available to evaluate the quality of care provided in maternity hospitals and will allow us to verify differences in the quality of data recording between cases and controls and among maternities according to their structural resources.

## Conclusion

The *Severe Maternal Morbidity* and *Perinatal Mortality* studies is expected to increase the knowledge about the magnitude and causes of perinatal deaths, severe maternal morbidity and maternal near miss, as well as their determinants in nationally representative hospitals and maternities. The results will allow us to identify areas of greater vulnerability and subsidize the development of public policies to reorganize the delivery and birth care network in Brazil.
